# Supplementary material for: Mitotic microhomology-mediated break-induced replication promotes chromoanasynthesis
Source: Nat Commun. 2026 Mar 3;17:3375. doi: 10.1038/s41467-026-70086-y (PMC13065848; doi:10.1038/s41467-026-70086-y)
Supplement: Supplementary file 1 — Supplementary Information [file 41467_2026_70086_MOESM1_ESM.pdf]

Supplementary Information

Mitotic microhomology-mediated break-induced replication promotes chromoanasynthesis

Greg H.P. Ngo[1]*, Kez Cleal[1], Sara Seifan[1], Vanda Miklos[1], Szymon A. Barwacz[2], Brian L. Ruis[3], Siamak A. Kamranvar[1], Julia W. Grimstead[1], Ying Liu[2], Eric A. Hendrickson[3], Duncan M. Baird[1]*.

[1]Division of Cancer and Genetics, School of Medicine, Cardiff University, Heath Park, Cardiff, CF14 4XN, UK.

[2]Center for Chromosome Stability, Department of Cellular and Molecular Medicine, University of Copenhagen; Blegdamsvej 3B, 2200, Copenhagen, Denmark.

[3]Department of Medicine, University of Virginia; Charlottesville, VA 22903, USA.

Corresponding author: NgoG@cardiff.ac.uk (G.N.); bairddm@cardiff.ac.uk (D.B.)

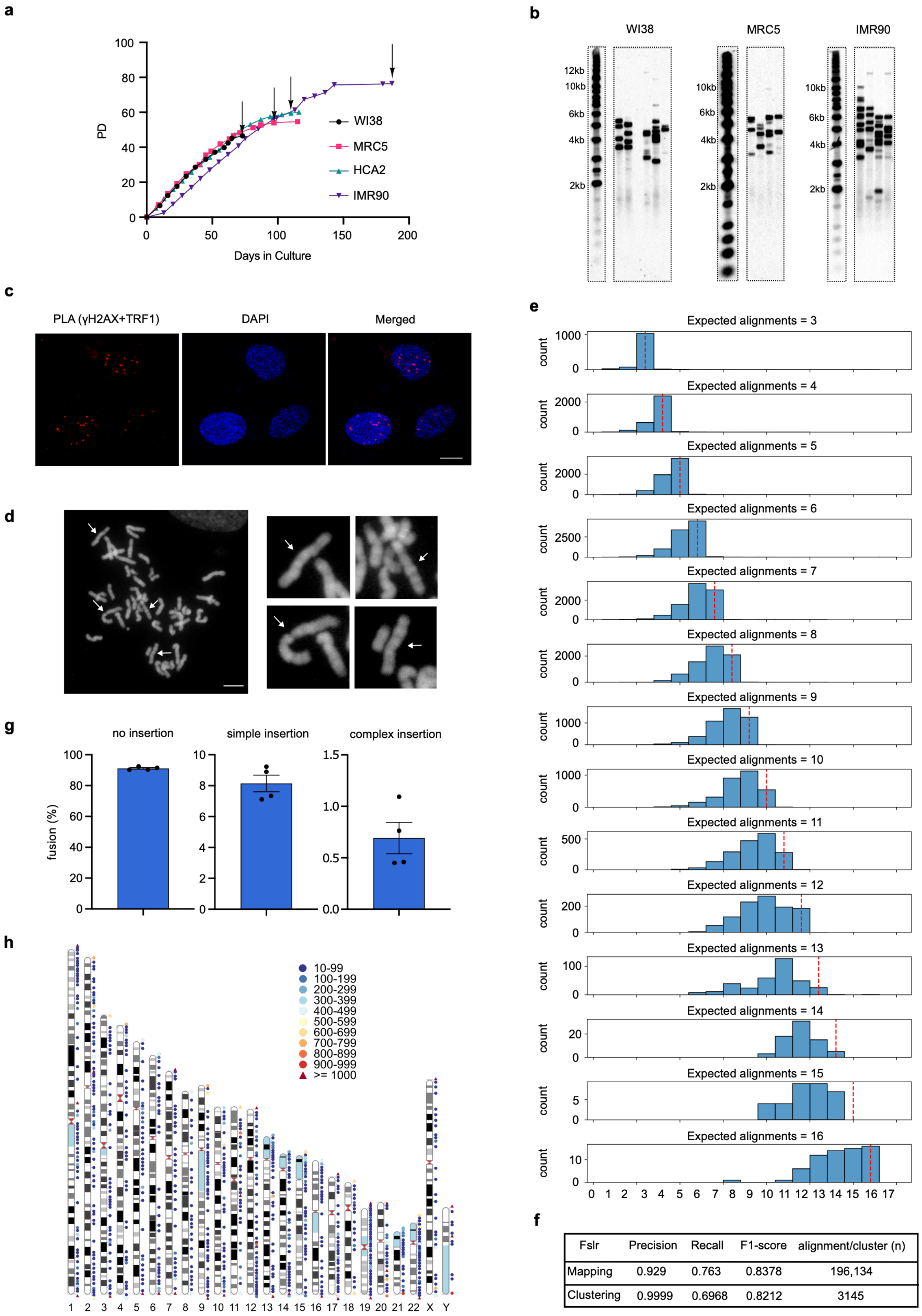

Fslr	Precision	Recall	F1-score	alignment/cluster (n)
Mapping	0.929	0.763	0.8378	196,134
Clustering	0.9999	0.6968	0.8212	3145

Supplementary Fig. 1. Fusion-seq long-read (FSLR) uncovers CCR in telomere crisis.

a Growth curves of four primary human fibroblasts transiting telomere crisis. Cells were harvested for telomere fusion analysis at the point indicated by the arrows. **b** Telomere fusions amplified using 17p6, XpYpM and 21q1 PCR primers from WI38, MRC5 and IMR90 fibroblast were subjected to Southern blot and detected using a 17p (shown here), XpYp or 21q telomere adjacent probe. Each lane represents separate PCR reaction containing 50 ng of template DNA from the indicated cell lines. This experiment was repeated independently with similar results for three times. **c** Proximity ligation assay (PLA) of γH2AX and TRF1 detected telomere dysfunction induced foci in IMR90 cells experiencing telomere crisis. Scale bars, 10 µm. This experiment was repeated independently with similar results for two times. **d.** Representative image of metaphase spread analysis of IMR90 cells experiencing telomere crisis. Arrows indicate the positions of the zoomed images where chromosome end-to-end fusions can be seen. Scale bars, 10 µm. This experiment was repeated independently with similar results for two times. **e** Histograms showing expected number of alignments versus recovered alignments using fslr. Telomere fusion events with 1 to 14 insertions (expected alignments 3 to 16) were simulated and the number of mapped alignments by the fslr pipeline was compared to the expected alignment number. The red dashed line indicates the expected number of alignments for an event and the blue bars show the number of fslr outputs with the indicated alignment number. The result shows that the pipeline either correctly maps or underestimates the alignment number but rarely over-estimates insertion complexity. **f** Benchmark results for mapping and clustering stages of fslr using simulated data. **g** Bar chart showing telomere fusion molecules with no insertion, simple (1 or 2) insertion or complex (3 or more) insertion in four human fibroblasts. Data plotted are means +/- standard error of the mean (s.e.m, n=4 biological replicates). **h** Karyotype plot showing the chromosomal location of insertions, with loci colour coded according to the number of insertions at each locus. Source data are provided as a Source Data file.

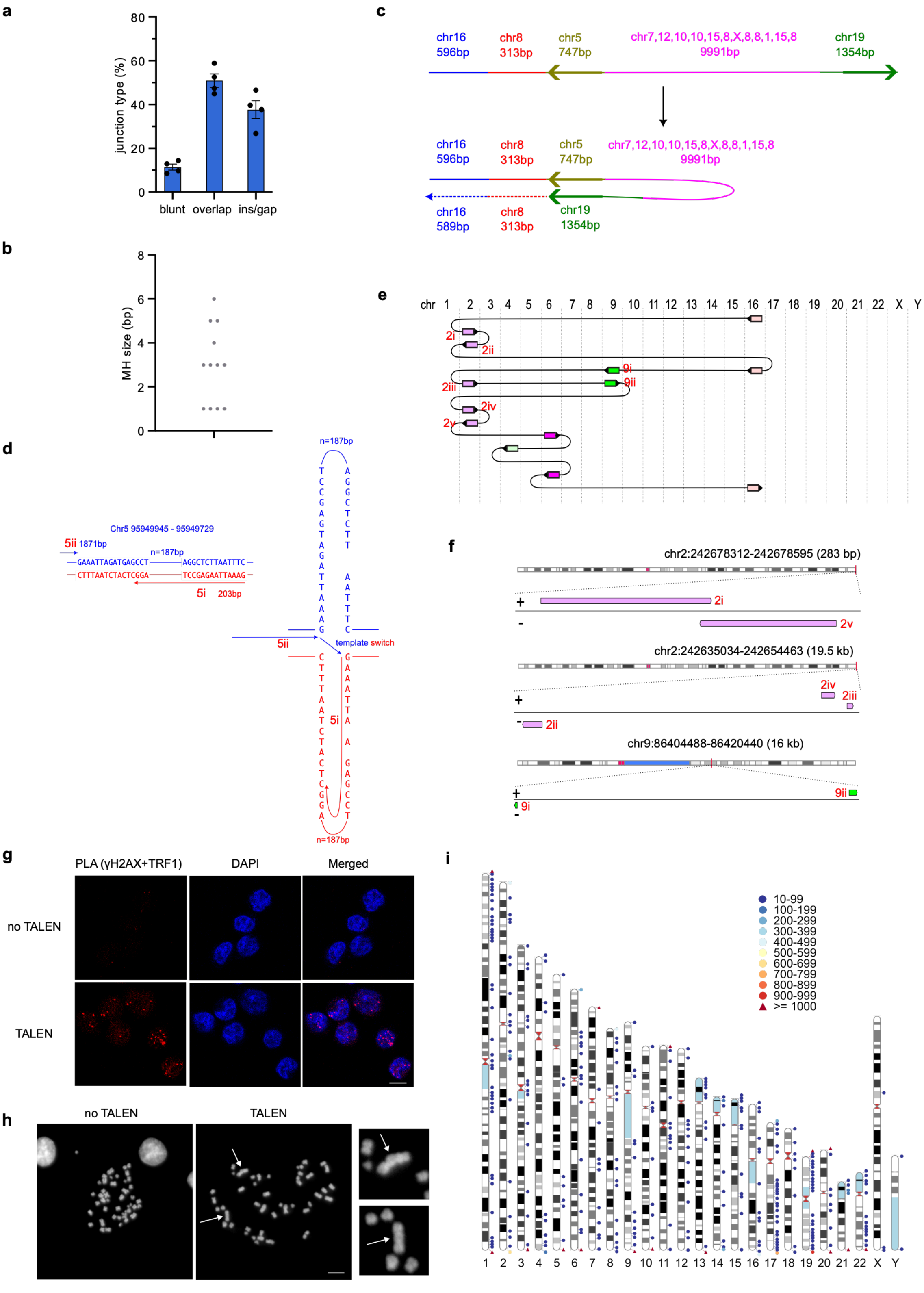
a
junction type (%)
blunt overlap ins/gap
b
MH size (bp)
c
chr16 596bp
chr8 313bp
chr5 747bp
chr7,12,10,10,15,8,X,8,8,1,15,8 9991bp
chr19 1354bp
chr16 589bp
d
Chr5 95949945 - 95949729
5ii 1871bp
n=187bp
5i 203bp
template switch
e
f
chr2:242678312-242678595 (283 bp)
chr2:242635034-242654463 (19.5 kb)
chr9:86404488-86420440 (16 kb)
g
PLA (γH2AX+TRF1)
DAPI
Merged
no TALEN
TALEN
h
no TALEN
TALEN
i
10-99
100-199
200-299
300-399
400-499
500-599
600-699
700-799
800-899
900-999
>= 1000

Supplementary Fig. 2. CCRs uncovered in telomere crisis and at sub-telomeric DNA double-strand breaks.

a Quantification of junction type in telomere fusion molecules derived from four human fibroblasts. Data plotted are means +/- standard error of the mean (s.e.m) (n = 4 biological replicates). **b** Scatter plot showing the size of microhomology (MH) at the junctions in complex 18. **c** Diagram showing how complex 18 could be generated by inverted repeat- (indicated by the arrows) driven foldback and DNA synthesis (indicated by dotted lines). **d** Diagram showing the generation of two insertions (highlighted with red text in Fig. 1j, 1k) by a template switching mechanism at an inverted repeat sequence. **e** Diagram showing how DNA molecules from different chromosomes are connected in complex 13. Direction of the arrows indicates the orientation of the DNA (right = +, left = -), chr = chromosome. **f** Genome browser (GW) plot showing the genomic location of selected DNA alignments from complex 13 (highlighted with red text in Supplementary Fig. 2e). **g** Proximity ligation assay (PLA) of γH2AX and TRF1 in HCT116 cells with or without TALEN transfection. Scale bars, 10 µm. This experiment was repeated independently with similar results for two times. **h** Representative image of metaphase spread analysis of HCT116 cells with or without TALEN transfection. Arrows indicate the positions of the zoomed images where chromosome end-to-end fusions can be seen. Scale bars, 10 µm. This experiment was repeated independently with similar results for two times. **i** Karyotype plot showing the chromosomal location of insertions from HCT116, with loci colour coded according to the number of insertions at each locus. Source data are provided as a Source Data file.

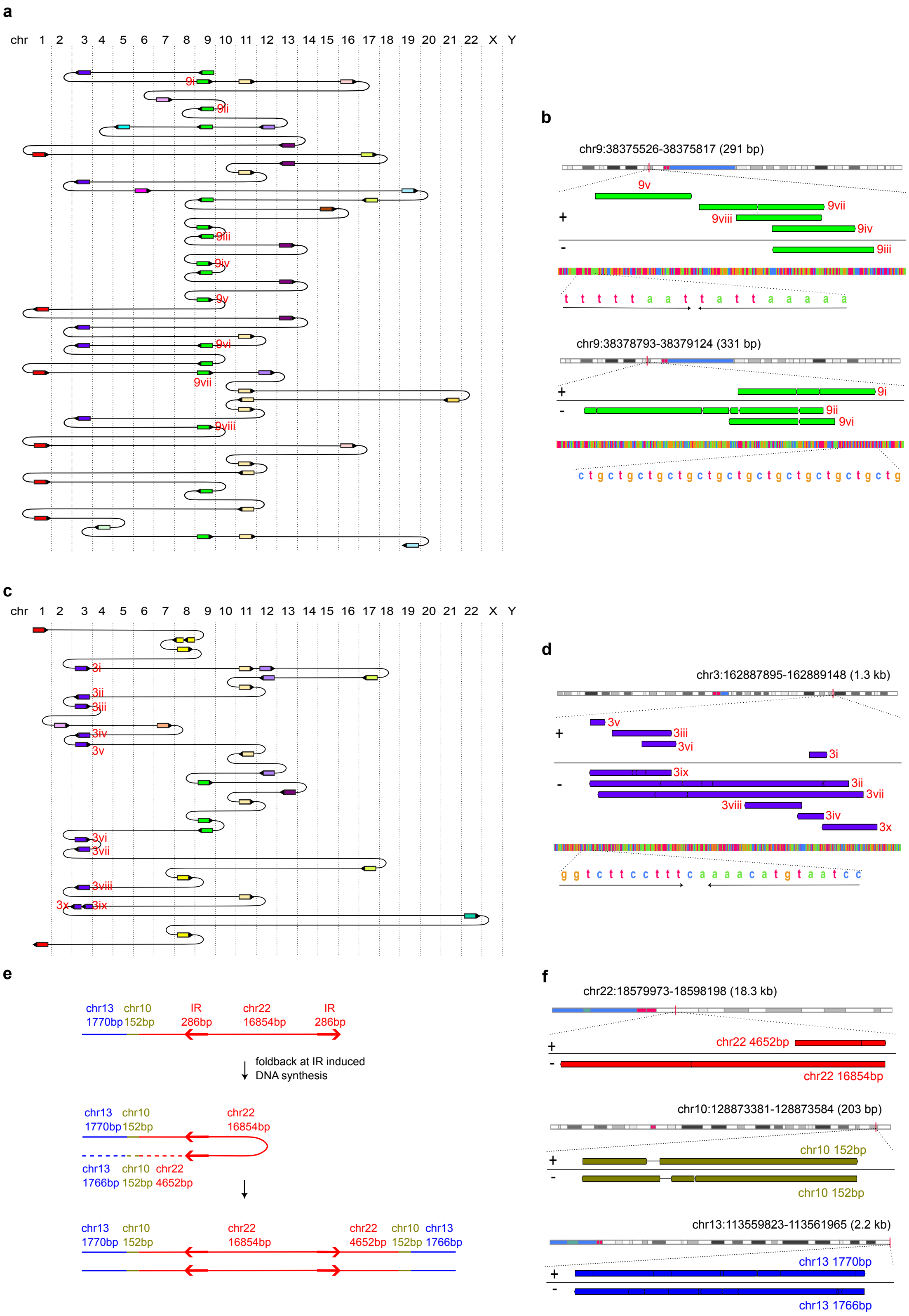

a
chr 1 2 3 4 5 6 7 8 9 10 11 12 13 14 15 16 17 18 19 20 21 22 X Y
9i
9ii
9iii
9iv
9v
9vi
9vii
9viii
b
chr9:38375526-38375817 (291 bp)
9v
9vii
9viii
9iv
9iii
chr9:38378793-38379124 (331 bp)
9i
9ii
9vi
c
chr 1 2 3 4 5 6 7 8 9 10 11 12 13 14 15 16 17 18 19 20 21 22 X Y
3i
3ii
3iii
3iv
3v
3vi
3vii
3viii
3x
3ix
d
chr3:162887895-162889148 (1.3 kb)
3v
3iii
3vi
3i
3ix
3ii
3vii
3viii
3iv
3x
e
chr13 1770bp
chr10 152bp
IR 286bp
chr22 16854bp
IR 286bp
foldback at IR induced DNA synthesis
chr13 1766bp
chr10 152bp
chr22 4652bp
chr22 4652bp
chr10 152bp
chr13 1766bp
f
chr22:18579973-18598198 (18.3 kb)
chr22 4652bp
chr22 16854bp
chr10:128873381-128873584 (203 bp)
chr10 152bp
chr10 152bp
chr13:113559823-113561965 (2.2 kb)
chr13 1770bp
chr13 1766bp

Supplementary Fig. 3. Sub-telomeric DNA double-strand breaks induce CCR with a replicative origin.

a,c Diagram showing how DNA molecules from different chromosomes are connected in complex 56 (a) and complex 34 (c), chr = chromosome. **b,d** Genome browser (GW) plot showing the location of each individual DNA alignment from complex 56 and complex 34 (highlighted with red text in Supplementary Fig. 3a, 3c) at their mapped genomic loci. The four-colour bar at the bottom of each panel represents the DNA sequences at the locus (red = T, blue = C, orange = G, green = A). Flanking DNA sequence predicted to a form secondary structure are indicated by lines with arrowheads at the bottom of each panel. **e** Diagram showing how complex 6 could be generated through an inverted repeat (IR, indicated by the arrows)- driven fold back and DNA synthesis (indicated by dotted lines). **f** Genome browser (GW) plot showing the location of each individual DNA alignment from complex 6 (Supplementary Fig. 3e) at their mapped genomic loci.

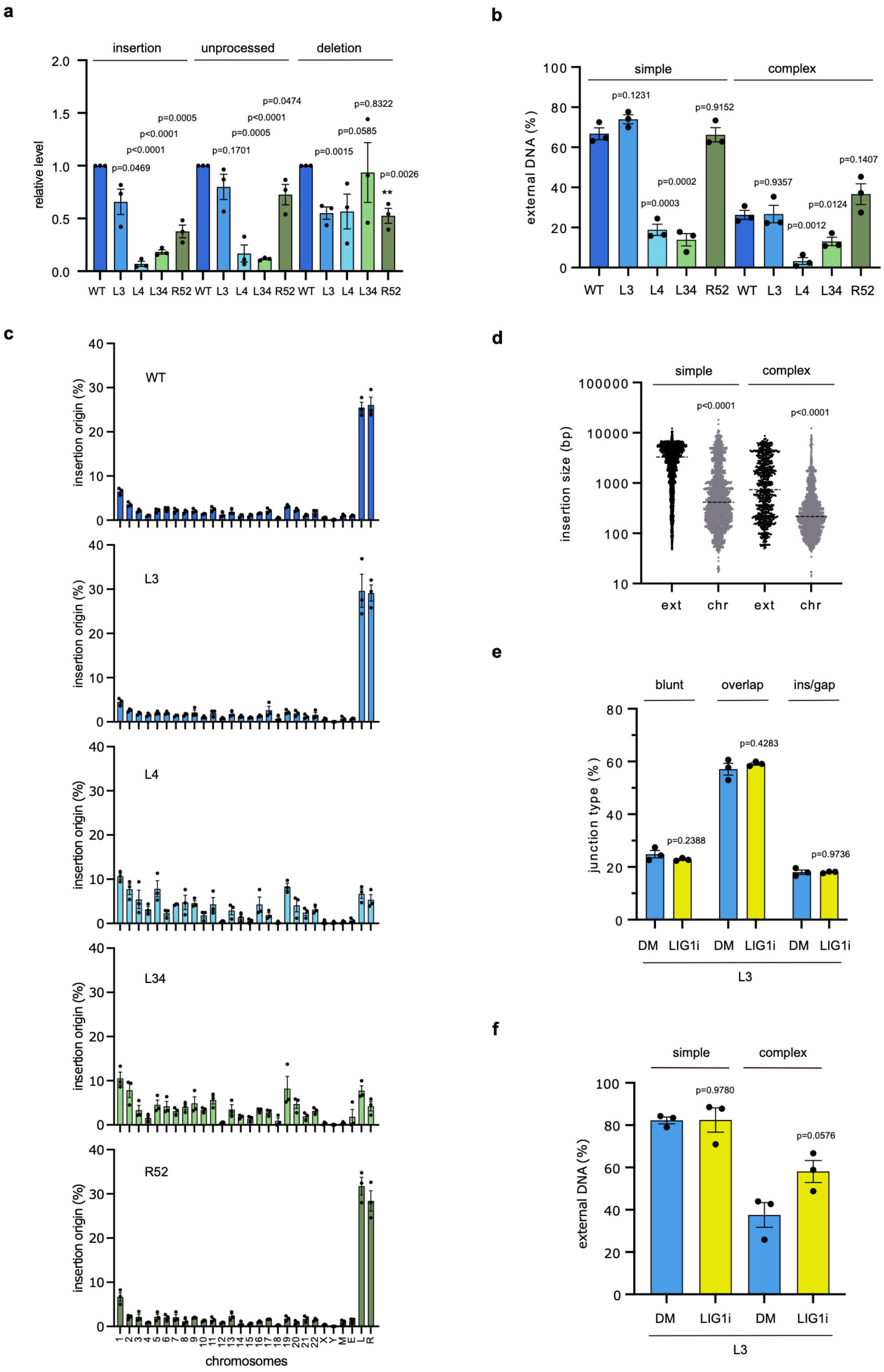
a
insertion
unprocessed
deletion
relative level
p=0.0469
p<0.0001
p<0.0001
p=0.0005
p=0.1701
p=0.0005
p<0.0001
p=0.0474
p=0.0015
p=0.0585
p=0.8322
p=0.0026
**
WT L3 L4 L34 R52 WT L3 L4 L34 R52 WT L3 L4 L34 R52
b
simple
complex
external DNA (%)
p=0.1231
p=0.0003
p=0.0002
p=0.9152
p=0.9357
p=0.0012
p=0.0124
p=0.1407
WT L3 L4 L34 R52 WT L3 L4 L34 R52
c
WT
L3
L4
L34
R52
insertion origin (%)
1 2 3 4 5 6 7 8 9 10 11 12 13 14 15 16 17 18 19 20 21 22 X Y M E L R
chromosomes
d
simple
complex
insertion size (bp)
p<0.0001
p<0.0001
ext chr ext chr
e
blunt
overlap
ins/gap
junction type (%)
p=0.2388
p=0.4283
p=0.9736
DM LIG1i DM LIG1i DM LIG1i
L3
f
simple
complex
external DNA (%)
p=0.9780
p=0.0576
DM LIG1i DM LIG1i
L3

Supplementary Fig. 4. CCRs arise independently of LIG4 but requires LIG1/3 and RAD52.

a Bar chart showing quantification of the intensity of fusion bands in HCT116 wild-type (WT) or *LIG3* KO (3), *LIG4* KO (4), *LIG3:LIG4* double KO (34) and *RAD52* KO (52) (relative to values in WT). Data plotted are means +/- s.e.m (n = 3 biological replicates). P values were obtained using Student's t-test (unpaired two-tailed, equal variance). **b** Quantification of the level of external DNA in simple (1 or 2 insertions) or complex (3 or more insertions) telomere fusion molecules isolated and amplified from HCT116 wild-type (WT) or *LIG3* KO (L3), *LIG4* KO (L4), *LIG3:LIG4* double KO (L34) and *RAD52* KO (R52). Data plotted are means +/- s.e.m (n = 3 biological replicates). P values were obtained as described in Supplementary Fig. 4a. **c** Quantification of the origin of individual DNA insertions in telomere fusion molecules isolated from HCT116 wild-type (WT) or *LIG3* KO (L3), *LIG4* KO (L4), *LIG3:LIG4* double KO (L34) and *RAD52* KO (R52). M= mitochondrial DNA, E= *E. coli* DNA, L= left TALEN and R= right TALEN plasmid. Data plotted are means +/- s.e.m (n = 3 biological replicates). **d** Scatter plot showing the size of individual DNA insertions from external (ext) or chromosomal DNA (chr) in simple (1 or 2 insertions) or complex (3 or more insertions) telomere fusion molecules from three biological replicates (n= 4214, 2117, 887, 2513 from left to right, dotted line = median). P values were obtained using two-tailed Mann-Whitney test. **e** Quantification of junction type in HCT116 *LIG3* KO (L3) treated with DMSO (DM) or LIG1 inhibitor (LIG1i). Data plotted are means +/- s.e.m (n=3 biological replicates). P values were obtained using Student's t-test (unpaired two-tailed, equal variance). **f** Quantification of the level of external DNA in simple (1 or 2 insertions) or complex (3 or more insertions) telomere fusion molecules isolated and amplified from HCT116 *LIG3* KO (L3) treated with DMSO (DM) or LIG1 inhibitor (LIG1i). Data plotted are means +/- s.e.m (n = 3 biological replicates). P values were obtained using Student's t-test (unpaired two-tailed, equal variance). Source data are provided as a Source Data file.

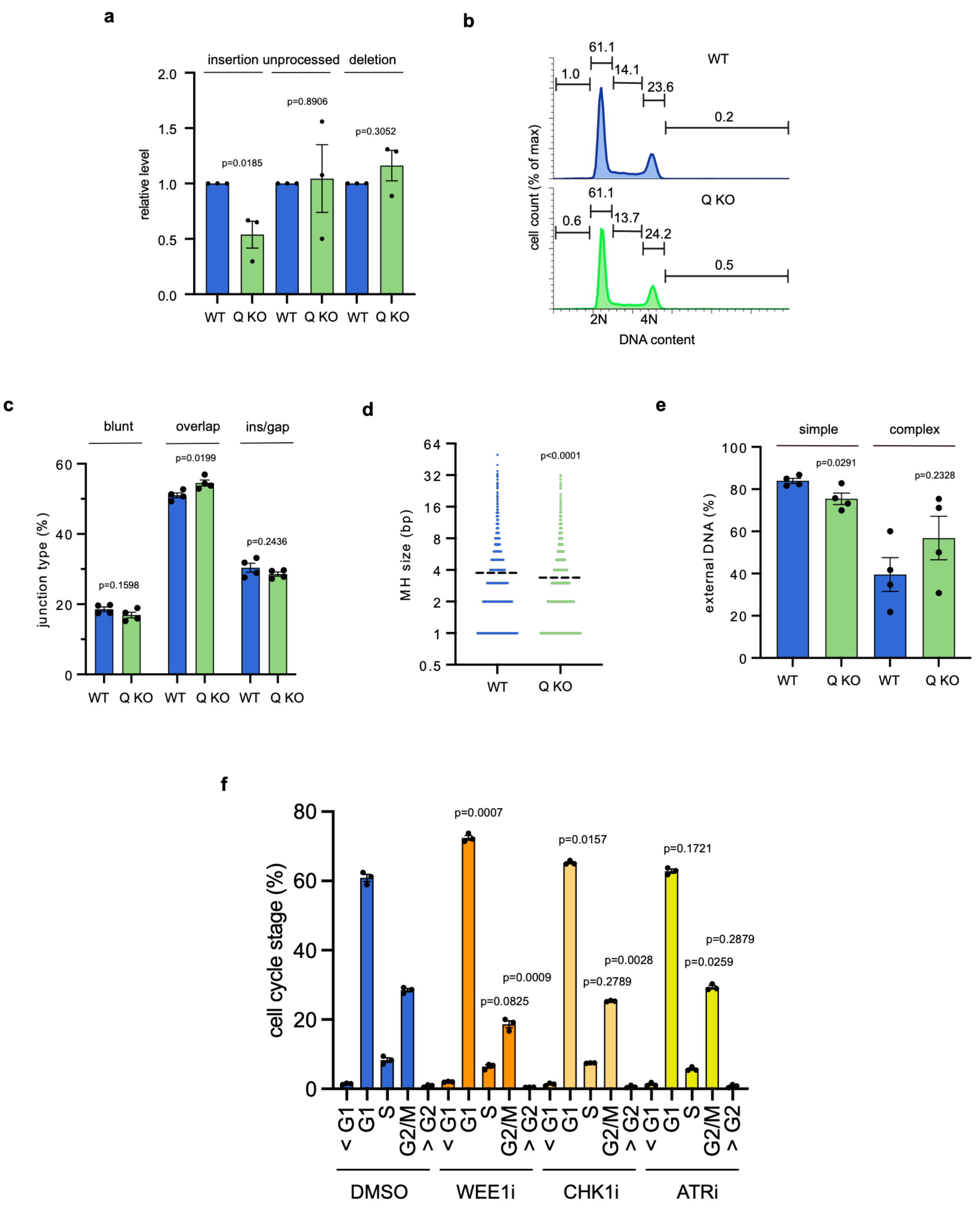
a
insertion
unprocessed
deletion
relative level
2.0
1.5
1.0
0.5
0.0
p=0.0185
p=0.8906
p=0.3052
WT
Q KO
WT
Q KO
WT
Q KO
b
WT
61.1
1.0
14.1
23.6
0.2
Q KO
61.1
0.6
13.7
24.2
0.5
cell count (% of max)
2N
4N
DNA content
c
blunt
overlap
ins/gap
junction type (%)
60
40
20
0
p=0.1598
p=0.0199
p=0.2436
WT
Q KO
WT
Q KO
WT
Q KO
d
MH size (bp)
64
32
16
8
4
2
1
0.5
p<0.0001
WT
Q KO
e
simple
complex
external DNA (%)
100
80
60
40
20
0
p=0.0291
p=0.2328
WT
Q KO
WT
Q KO
f
cell cycle stage (%)
80
60
40
20
0
p=0.0007
p=0.0825
p=0.0009
p=0.0157
p=0.2789
p=0.0028
p=0.1721
p=0.0259
p=0.2879
< G1
G1
S
G2/M
> G2
DMSO
WEE1i
CHK1i
ATRi

Supplementary Fig. 5. Chromoanasynthesis is promoted by Polθ during mitosis and suppressed by the DNA damage checkpoint.

a Bar chart showing quantification of the intensity of fusion bands in RPE1 WT and *POLQ* KO (Q KO) cells (relative to values in WT). Data plotted are means +/- s.e.m (n = 3 biological replicates). P values were obtained using Student's t-test (unpaired two-tailed, equal variance). **b** Representative plot showing the cell cycle distribution of RPE1 WT and *POLQ* KO (Q KO) cells. This experiment was repeated independently with similar results for three times. **c** Quantification of junction types in RPE1 WT and *POLQ* KO. Data plotted are means +/- s.e.m (n = 4 biological replicates). P values were obtained using Student's t-test (unpaired two-tailed, equal variance). **d** Scatter plot comparing the size of microhomology (MH) at junctions in RPE1 WT and *POLQ* KO (Q KO) cells from four biological replicates (n= 3043, 2779 from left to right, dotted line = means). P values were obtained using two-tailed Mann-Whitney test. **e** Quantification of the level of external DNA in simple (1 or 2 insertions) or complex (3 or more insertions) telomere fusion molecules isolated and amplified from RPE1 WT and *POLQ* KO cells. Data plotted are means +/- s.e.m (n = 4 biological replicates). P values were obtained using Student's t-test (unpaired two-tailed, equal variance). **f** Cell cycle analysis of RPE1 WT cells treated with DMSO, a WEE1 inhibitor (WEE1i), a CHK1 inhibitor (CHK1i) or an ATR inhibitor (ATRi) two days after TALEN nucleofection. Data plotted are means +/- s.e.m (n = 3 biological replicates) P values were obtained using Student's t-test (unpaired two-tailed, equal variance). Source data are provided as a Source Data file.

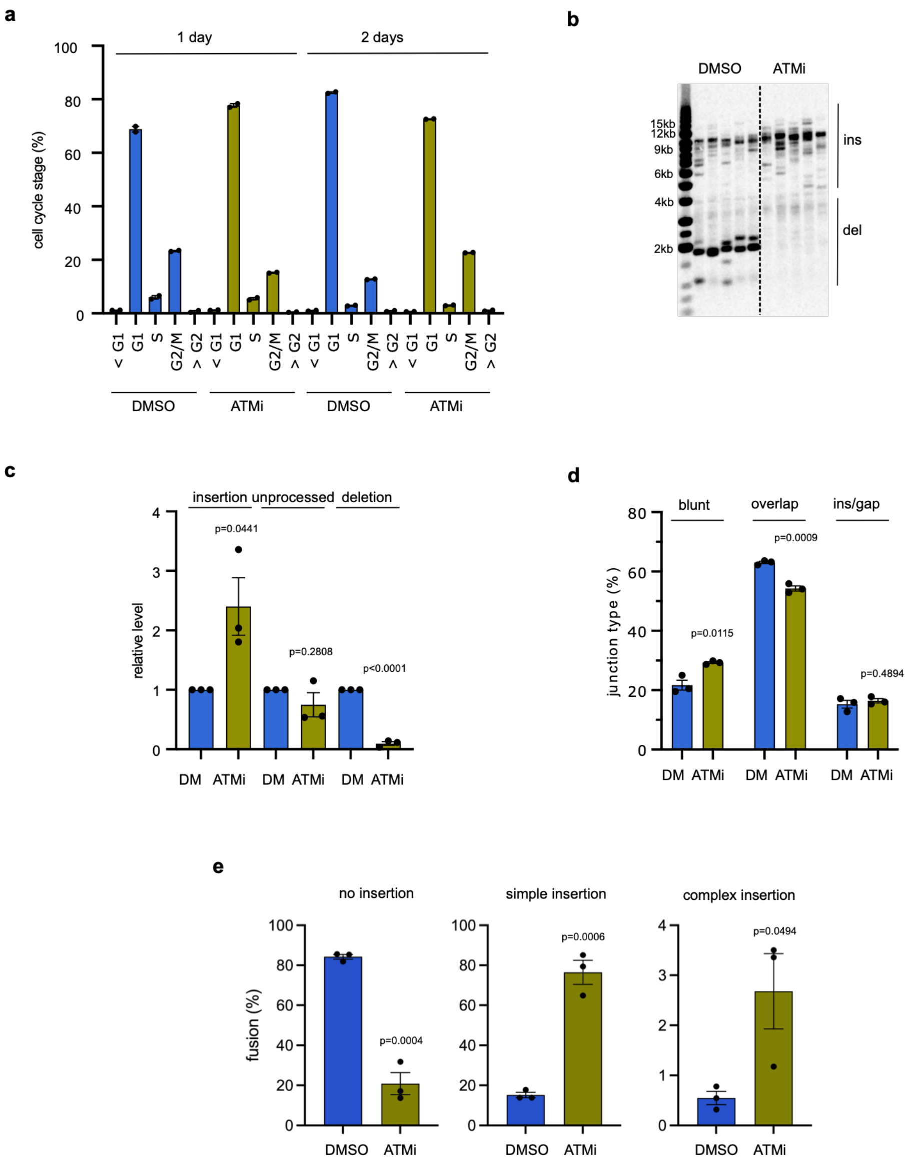
a
1 day
2 days
cell cycle stage (%)
100
80
60
40
20
0
< G1
G1
S
G2/M
> G2
DMSO
ATMi
DMSO
ATMi
b
DMSO
ATMi
15kb
12kb
9kb
6kb
4kb
2kb
ins
del
c
insertion unprocessed deletion
relative level
p=0.0441
p=0.2808
p<0.0001
4
3
2
1
0
DM ATMi DM ATMi DM ATMi
d
blunt
overlap
ins/gap
junction type (%)
p=0.0115
p=0.0009
p=0.4894
80
60
40
20
0
DM ATMi DM ATMi DM ATMi
e
no insertion
simple insertion
complex insertion
fusion (%)
p=0.0004
p=0.0006
p=0.0494
DMSO
ATMi

Supplementary Fig. 6. CCRs are suppressed by ATM.

a Cell cycle analysis of RPE1 WT cells treated with DMSO, or an ATM inhibitor (ATMi) one or two days after TALEN nucleofection. Data plotted are means +/- s.e.m (n = 2 biological replicates). **b** Telomere fusion blot showing telomere fusion molecules amplified from RPE1 WT cells treated with DMSO, or an ATM inhibitor (ATMi), two days after TALEN nucleofection (ins = insertion, del = deletion). This experiment was repeated independently with similar results for three times**. c** Bar chart showing quantification of the intensity of fusion bands in RPE1 WT cells treated with DMSO, or an ATM inhibitor (ATMi) (relative to values in DMSO). Data plotted are means +/- s.e.m (n = 3 biological replicates). P values were obtained using Student's t-test (unpaired two-tailed, equal variance). **d** Quantification of junction types in RPE1 WT cells treated with DMSO, or an ATM inhibitor (ATMi). Data plotted are means +/- s.e.m (n = 3 biological replicates). P values were obtained using Student's t-test (unpaired two-tailed, equal variance)**. e** Bar chart comparing telomere fusion molecules with no insertion, simple (1 or 2) insertion or complex (3 or more) insertion in RPE1 WT cells treated with DMSO or an ATM inhibitor (ATMi). Data plotted are means +/- s.e.m (n=3 biological replicates). P values were obtained using Student's t-test (unpaired two-tailed, equal variance). Source data are provided as a Source Data file.

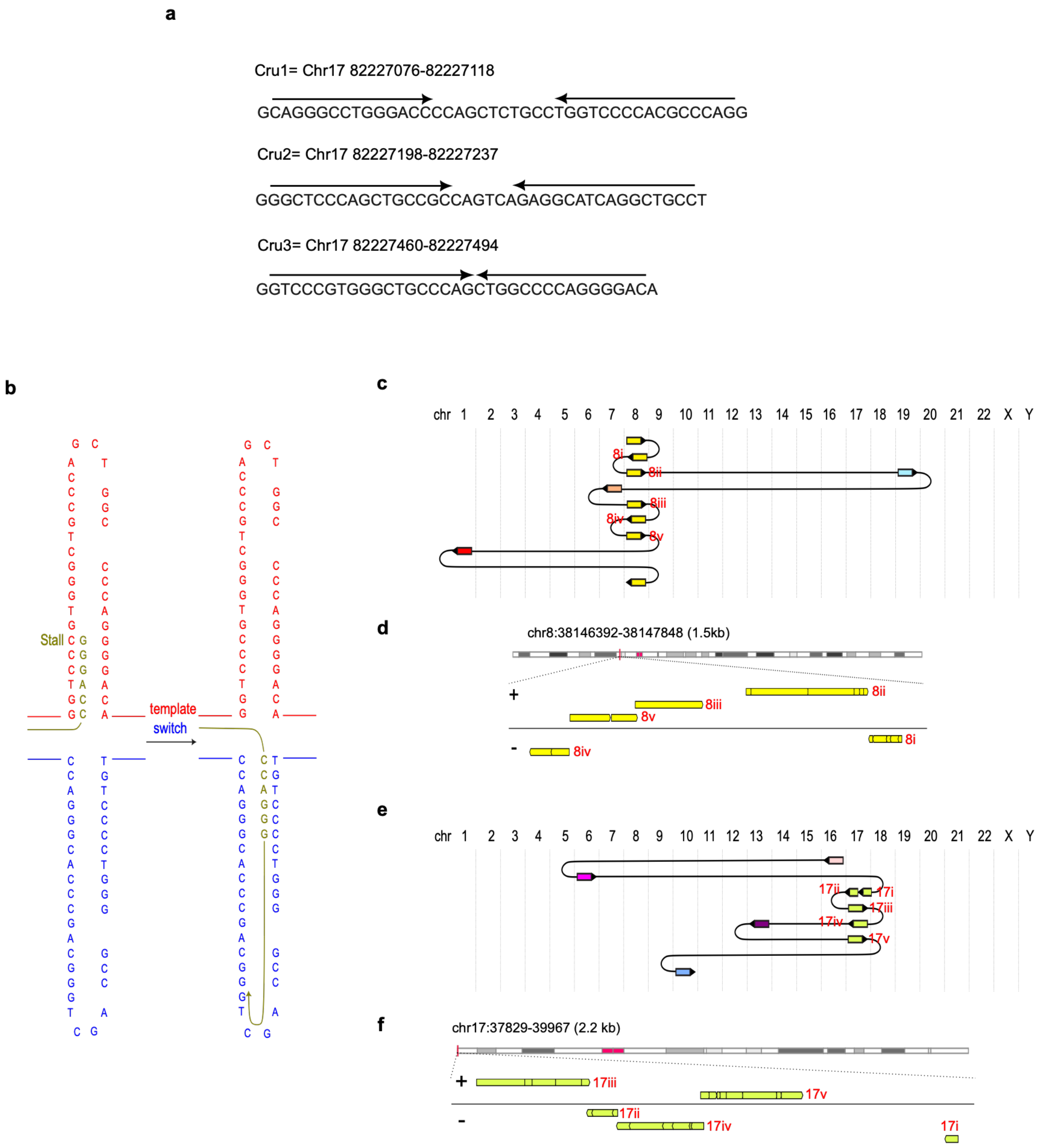
a
Cru1= Chr17 82227076-82227118
GCAGGGCCTGGGACCCCAGCTCTGCCTGGTCCCCACGCCCAGG
Cru2= Chr17 82227198-82227237
GGGCTCCCAGCTGCCGCCAGTCAGAGGCATCAGGCTGCCT
Cru3= Chr17 82227460-82227494
GGTCCCGTGGGCTGCCCAGCTGGCCCCAGGGGACA
b
Stall
template
switch
c
chr 1 2 3 4 5 6 7 8 9 10 11 12 13 14 15 16 17 18 19 20 21 22 X Y
8i
8ii
8iii
8iv
8v
d
chr8:38146392-38147848 (1.5kb)
+
8ii
8iii
8v
-
8i
8iv
e
chr 1 2 3 4 5 6 7 8 9 10 11 12 13 14 15 16 17 18 19 20 21 22 X Y
17ii
17i
17iii
17iv
17v
f
chr17:37829-39967 (2.2 kb)
+
17iii
17v
-
17ii
17iv
17i

Supplementary Fig. 7. CCRs are suppressed by the DNA damage checkpoint.

a Three inverted repeat DNA sequences (indicated by arrows) from complex 134 which can generate cruciform structures to induce template switching. **b** Possible mechanism of how Polθ could stimulate inverted repeat-induced template switching by promoting the annealing of dissociated single-stranded DNA to the opposite side of the cruciform structure. **c** Diagram showing how DNA molecules from different chromosomes are connected in complex 10, which was isolated from RPE1 WT cells treated with the WEE1 inhibitor. Direction of the arrows indicate the orientation of the DNA (right = +, left = -), chr = chromosome. **d** Genome browser (GW) plot showing the genomic location of individual DNA alignment from complex 10 (highlighted with red text in Supplementary Fig.7c). **e** Diagram showing how DNA molecules from different chromosomes are connected in complex 9, isolated from RPE1 WT cell treated with the WEE1 inhibitor. Direction of arrows indicate the orientation of the DNA (right = +, left = -), chr = chromosome. **f** Genome browser (GW) plot showing the location of each individual DNA alignment from complex 9 (highlighted with red text in Supplementary Fig. 7e) at their mapped genomic loci.

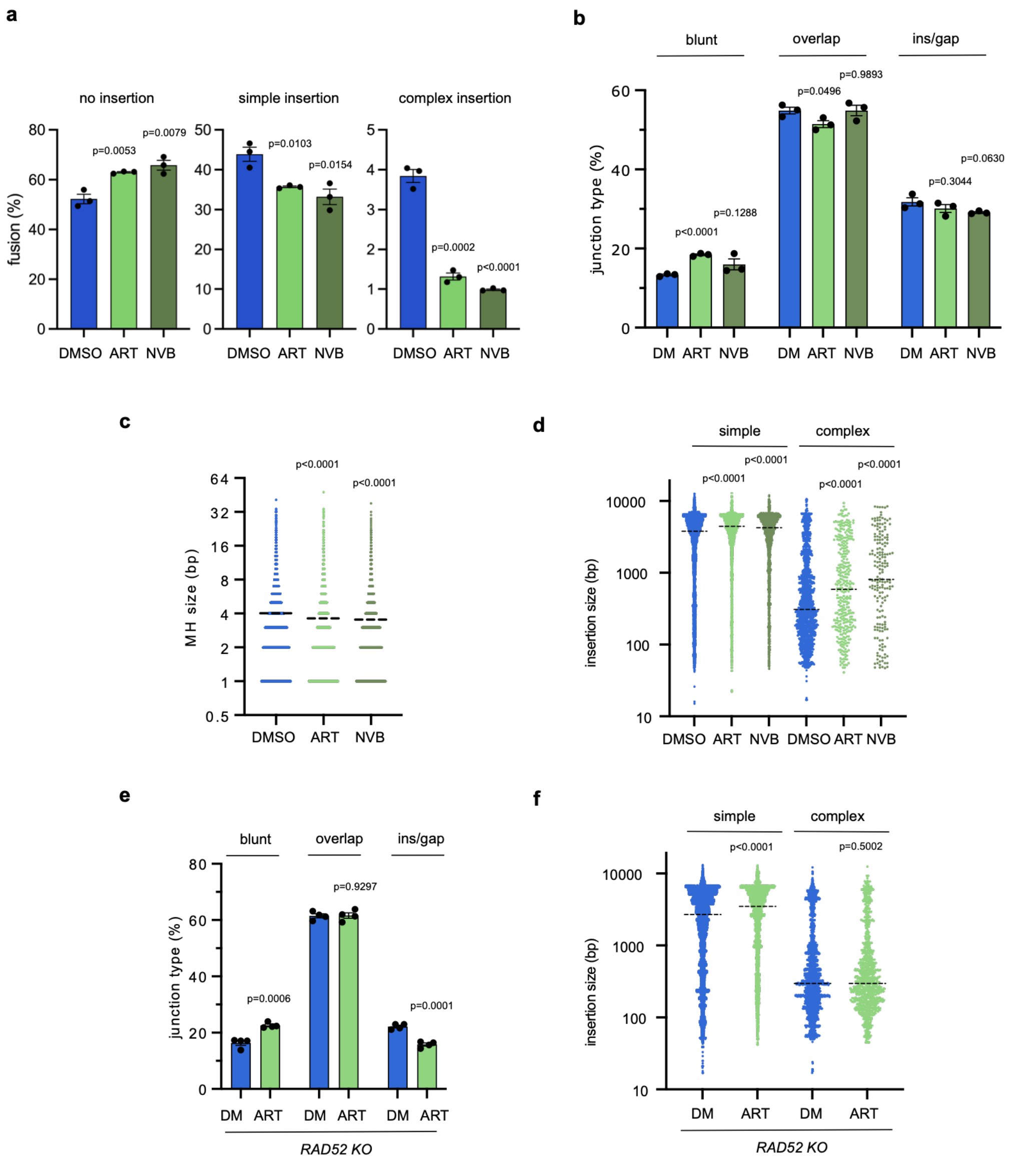
a
no insertion
simple insertion
complex insertion
fusion (%)
p=0.0053
p=0.0079
p=0.0103
p=0.0154
p=0.0002
p<0.0001
DMSO ART NVB
b
blunt
overlap
ins/gap
junction type (%)
p<0.0001
p=0.1288
p=0.0496
p=0.9893
p=0.3044
p=0.0630
DM ART NVB
c
MH size (bp)
p<0.0001
p<0.0001
DMSO ART NVB
d
simple
complex
insertion size (bp)
p<0.0001
p<0.0001
p<0.0001
p<0.0001
DMSO ART NVB DMSO ART NVB
e
blunt
overlap
ins/gap
junction type (%)
p=0.0006
p=0.9297
p=0.0001
DM ART DM ART DM ART
RAD52 KO
f
simple
complex
insertion size (bp)
p<0.0001
p=0.5002
DM ART DM ART
RAD52 KO

Supplementary Fig. 8. Chromoanasynthesis at dysfunctional telomeres can be reduced by Polθ inhibitors.

a Bar chart comparing telomere fusion molecules with no insertion, simple (1 or 2) insertion or complex (3 or more) insertion in RPE1 WT treated with WEE1 inhibitors with or without Polθ inhibitors (ART = ART558, NVB = novobiocin). Data plotted are means +/- s.e.m (n=3 biological replicates). P values were obtained using Student's t-test (unpaired two-tailed, equal variance). **b** Quantification of the junction types in RPE1 WT treated with WEE1 inhibitors with or without Polθ inhibitors (ART = ART558, NVB = novobiocin). Data plotted are means +/- s.e.m (n = 3 biological replicates). P values were obtained using Student's t-test (unpaired two-tailed, equal variance). **c** Scatter plot comparing the size of microhomology (MH) at junctions in RPE1 WT cells treated with WEE1 inhibitors with or without Polθ inhibitors (ART = ART558, NVB = novobiocin) from three biological replicates (n= 8181, 4178, 3165 from left to right, dotted line = means) P values were obtained using two-tailed Mann-Whitney test. **d** Scatter plot showing the size of individual DNA insertions in simple (1 or 2 insertions) or complex (3 or more insertions) telomere fusion molecules from three biological replicates (n= 5649, 3060, 2163, 1522, 354, 186 from left to right, dotted line = median). P values were obtained using two-tailed Mann-Whitney test. **e** Quantification of the junction types in HCT116 *RAD52* KO treated with DMSO (DM) or Polθ inhibitor (ART558). Data plotted are means +/- s.e.m (n = 4 biological replicates). P values were obtained using Student's t-test (unpaired two-tailed, equal variance). **f** Scatter plot showing the size of individual DNA insertions in simple (1 or 2 insertions) or complex (3 or more insertions) telomere fusion molecules from four biological replicates (n= 10739, 7014, 2269, 1096 from left to right, dotted line = median) isolated and amplified from HCT116 *RAD52* KO treated with DMSO (DM) or Polθ inhibitor (ART558). P values were obtained using two-tailed Mann-Whitney test. Source data are provided as a Source Data file.

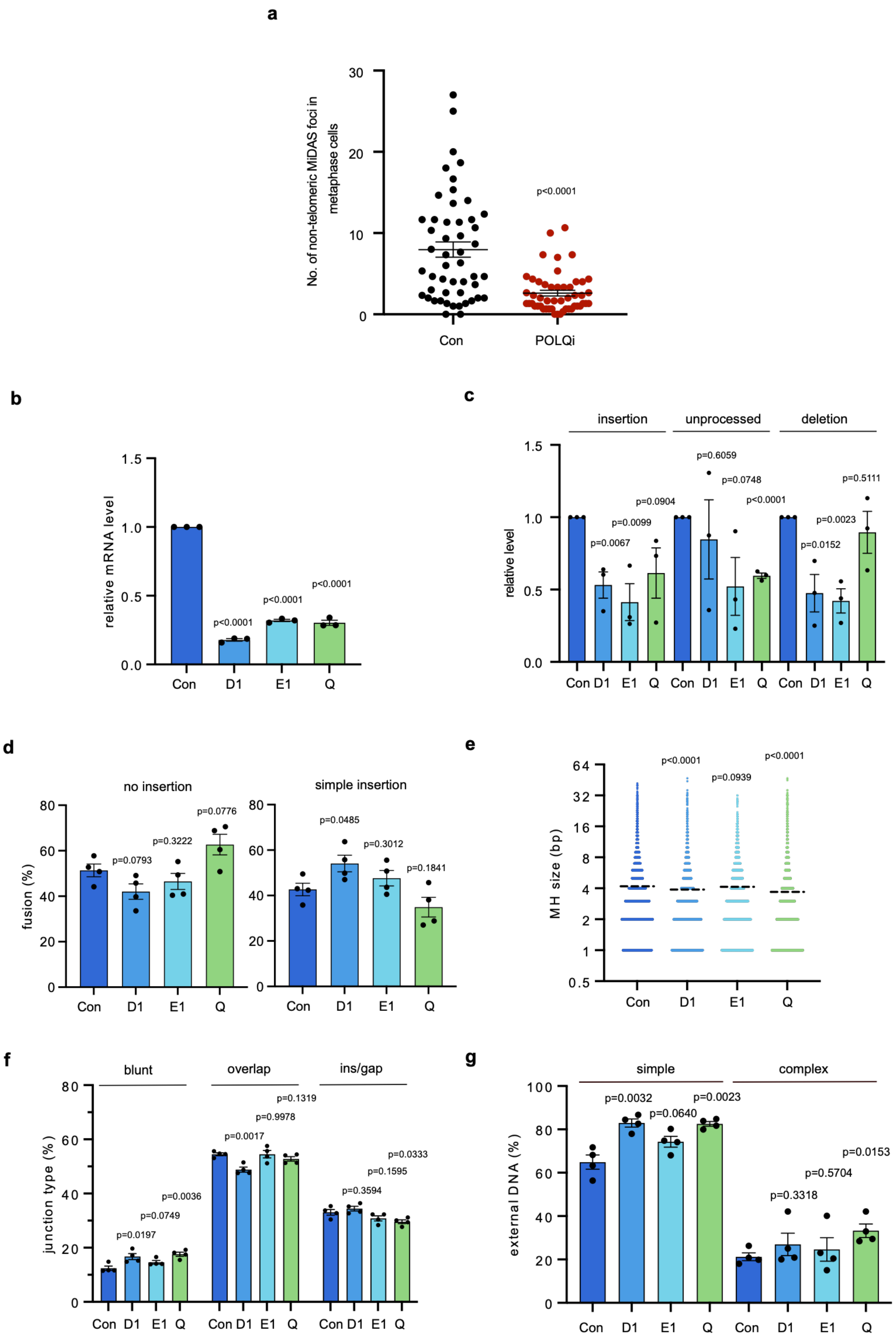
a
No. of non-telomeric MiDAS foci in metaphase cells
30
20
10
0
p<0.0001
Con
POLQi
b
relative mRNA level
1.5
1.0
0.5
0.0
p<0.0001
p<0.0001
p<0.0001
Con D1 E1 Q
c
insertion
unprocessed
deletion
relative level
1.5
1.0
0.5
0.0
p=0.0067
p=0.0099
p=0.0904
p=0.6059
p=0.0748
p<0.0001
p=0.0152
p=0.0023
p=0.5111
Con D1 E1 Q Con D1 E1 Q Con D1 E1 Q
d
no insertion
simple insertion
fusion (%)
80
60
40
20
0
p=0.0793
p=0.3222
p=0.0776
p=0.0485
p=0.3012
p=0.1841
Con D1 E1 Q Con D1 E1 Q
e
MH size (bp)
64
32
16
8
4
2
1
0.5
p<0.0001
p=0.0939
p<0.0001
Con D1 E1 Q
f
blunt
overlap
ins/gap
junction type (%)
80
60
40
20
0
p=0.0197
p=0.0749
p=0.0036
p=0.0017
p=0.9978
p=0.1319
p=0.3594
p=0.1595
p=0.0333
Con D1 E1 Q Con D1 E1 Q Con D1 E1 Q
g
simple
complex
external DNA (%)
100
80
60
40
20
0
p=0.0032
p=0.0640
p=0.0023
p=0.3318
p=0.5704
p=0.0153
Con D1 E1 Q Con D1 E1 Q

Supplementary Fig. 9. Mitotic MM-BIR requires Polδ.

a Quantification of non-telomeric MiDAS foci in metaphase cell treated as shown in Fig.5d. In each quantification, data are presented as mean +/- s.e.m of 3 independent experiments. In each replicate, 50 metaphase cells were analyzed in each condition. In total, 150 cells were analyzed for each condition (n=150). Statistical values were calculated with a two-tailed Mann–Whitney test. **b** RT-qPCR analysis showing the mRNA levels of *POLD1*, *POLE1* and *POLQ* in cells transfected with the respective siRNA relative to control. Data plotted are means +/- s.e.m (n = 3 biological replicates). P values were obtained using Student's t-test (unpaired two-tailed, equal variance). **c** Bar chart showing quantification of the intensity of fusion bands in RPE1 wild-type (WT) cells transfected with control siRNA (Con) or siRNA targeting *POLD1* (D1), *POLE1* (E1) or *POLQ* (Q) (relative to values in Con). Data plotted are means +/- s.e.m (n = 3 biological replicates). P values were obtained using Student's t-test (unpaired two-tailed, equal variance). **d** Bar chart comparing telomere fusion molecules. Data plotted are means +/- s.e.m (n=4 biological replicates). P values were obtained using Student's t-test (unpaired two-tailed, equal variance). **e** Scatter plot comparing the size of microhomology (MH) at junctions from four biological replicates (n= 14027, 4737, 3924, 6305 from left to right, dotted line = means). P values were obtained using a two-tailed Mann–Whitney test. **f** Quantification of junction type. Data plotted are means +/- s.e.m (n = 4 biological replicates). P values were obtained using Student's t-test (unpaired two-tailed, equal variance). **g** Quantification of the level of external DNA in simple (1 or 2 insertions) or complex (3 or more insertions) telomere fusion molecules isolated and amplified from RPE1 WT transfected with the indicated siRNA. Data plotted are means +/- s.e.m (n = 4 biological replicates). P values were obtained using Student's t-test (unpaired two-tailed, equal variance). Source data are provided as a Source Data file.

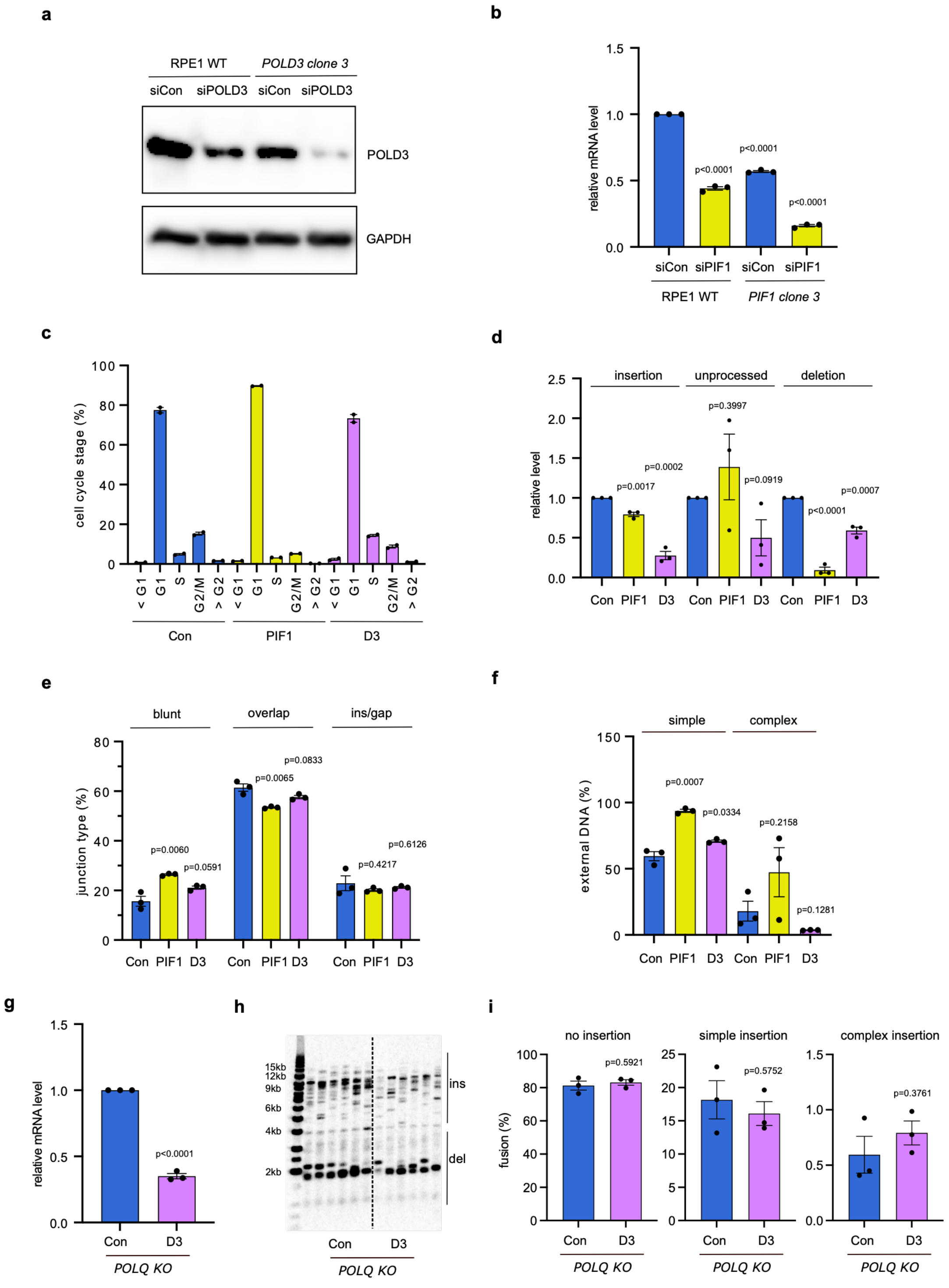
a
RPE1 WT
POLD3 clone 3
siCon
siPOLD3
siCon
siPOLD3
POLD3
GAPDH
b
relative mRNA level
1.5
1.0
0.5
0.0
p<0.0001
p<0.0001
p<0.0001
siCon
siPIF1
siCon
siPIF1
RPE1 WT
PIF1 clone 3
c
cell cycle stage (%)
100
80
60
40
20
0
< G1
G1
S
G2/M
> G2
Con
PIF1
D3
d
relative level
2.5
2.0
1.5
1.0
0.5
0.0
insertion
unprocessed
deletion
p=0.0017
p=0.0002
p=0.3997
p=0.0919
p<0.0001
p=0.0007
Con PIF1 D3 Con PIF1 D3 Con PIF1 D3
e
junction type (%)
80
60
40
20
0
blunt
overlap
ins/gap
p=0.0060
p=0.0591
p=0.0065
p=0.0833
p=0.4217
p=0.6126
Con PIF1 D3 Con PIF1 D3 Con PIF1 D3
f
external DNA (%)
150
100
50
0
simple
complex
p=0.0007
p=0.0334
p=0.2158
p=0.1281
Con PIF1 D3 Con PIF1 D3
g
relative mRNA level
1.5
1.0
0.5
0.0
p<0.0001
Con
D3
POLQ KO
h
15kb
12kb
9kb
6kb
4kb
2kb
ins
del
Con
D3
POLQ KO
i
no insertion
simple insertion
complex insertion
fusion (%)
100
80
60
40
20
0
p=0.5921
25
20
15
10
5
0
p=0.5752
1.5
1.0
0.5
0.0
p=0.3761
Con D3
POLQ KO

Supplementary Fig.10. Mitotic MM-BIR are regulated by PIF1 and POLD3.

a Western blot showing the level of POLD3 and GAPDH in RPE1 WT or *POLD3* heterozygote clone 3 transfected with Con or *POLD3* siRNA. This experiment was repeated independently with similar results for two times. **b** RT-qPCR analysis showing the mRNA levels of *PIF1* in RPE1 WT or *PIF1* KO clone 3 transfected with Con or *PIF1* siRNA. Data plotted are means +/- s.e.m (n = 3 biological replicates). P values were obtained using Student's t-test (unpaired two-tailed, equal variance). **c** Cell cycle analysis of RPE1 WT cells transfected with control siRNA (Con), *PIF1* KO cells transfected with *PIF1* siRNA (PIF1) and *POLD3* heterozygote cells transfected with *POLD3* siRNA (D3), two days after TALEN nucleofection. Data plotted are means +/- s.e.m (n = 2 biological replicates). **d** Bar chart showing quantification of the intensity of fusion bands in RPE1 WT cells transfected with control siRNA (Con), *PIF1* KO transfected with *PIF1* siRNA (PIF1) and *POLD3* heterozygote transfected with *POLD3* siRNA (D3) (relative to values in Con). Data plotted are means +/- s.e.m (n = 3 biological replicates). P values were obtained as described in Supplementary Fig.10b. **e** Quantification of junction type. Data plotted are means +/- s.e.m (n = 3 biological replicates). P values were obtained as described in Supplementary Fig.10b. **f** Quantification of the level of external DNA. Data plotted are means +/- s.e.m (n = 3 biological replicates). P values were obtained as described in Supplementary Fig.10b. **g** RT-qPCR analysis showing the mRNA levels of *POLD3* in RPE1 *POLQ* KO transfected with Con or *POLD3* siRNA. Data plotted are means +/- s.e.m (n = 3 biological replicates). P values were obtained as described in Supplementary Fig.10b. **h** Telomere fusion blot showing telomere fusion molecules amplified from RPE1 *POLQ* KO cells transfected with Con or *POLD3* siRNA (ins = insertion, del = deletion). This experiment was repeated independently with similar results for three times. **i** Bar chart comparing telomere fusion molecules. Data plotted are means +/- s.e.m (n=3 biological replicates). P values were obtained as described in Supplementary Fig.10b. Source data are provided as a Source Data file.

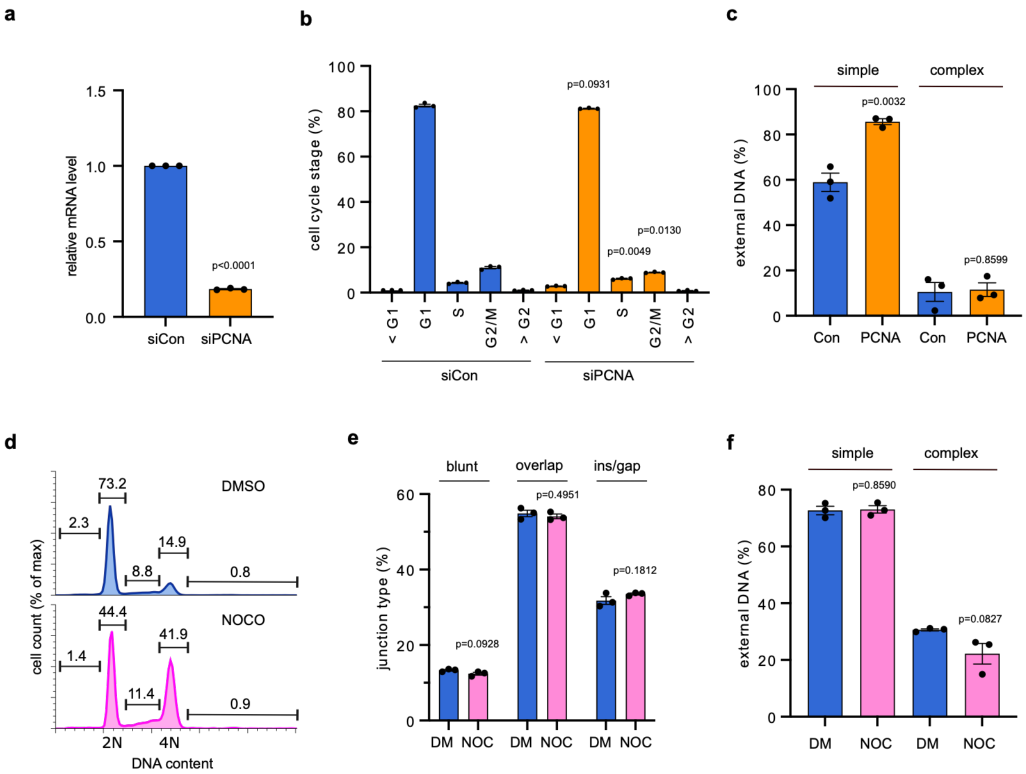

Supplementary Fig.11. Mitotic MM-BIR are regulated by PCNA and occur in early mitosis.

a RT-qPCR analysis showing the mRNA levels of *PCNA* (relative to control) in RPE1 WT cells transfected with control or *PCNA* siRNA. Data plotted are means +/- s.e.m (n = 3 biological replicates). P values were obtained using Student's t-test (unpaired two-tailed, equal variance). **b** Cell cycle analysis of RPE1 WT cells transfected with control or *PCNA* siRNA, two days after TALEN nucleofection. Data plotted are means + s.e.m (n = 3 biological replicates) P values were obtained using Student's t-test (unpaired two-tailed, equal variance). **c** Quantification of the level of external DNA in simple (1 or 2 insertions) or complex (3 or more insertions) telomere fusion molecules isolated and amplified from RPE1 WT cells transfected with control or *PCNA* siRNA. Data plotted are means +/- s.e.m (n = 3 biological replicates). P values were obtained using Student's t-test (unpaired two-tailed, equal variance). **d** Representative plot showing cell cycle distribution of RPE1 WT cells treated with a WEE1 inhibitor together with DMSO or nocodazole (NOCO) two days after TALEN nucleofection. This experiment was repeated independently with similar results for three times. **e** Quantification of junction type in RPE1 WT cells treated with a WEE1 inhibitor together with DMSO (DM) or nocodazole (NOCO). Data plotted are means +/- s.e.m (n = 3 biological replicates). DMSO controls were the same as in Supplementary Fig. 8a-d P values were obtained using Student's t-test (unpaired two-tailed, equal variance). **f** Quantification of the level of external DNA in simple (1 or 2 insertions) or complex (3 or more insertions) telomere fusion molecules isolated and amplified from RPE1 WT cells treated with a WEE1 inhibitor together with DMSO (DM) or nocodazole (NOC). Data plotted are means +/- s.e.m (n = 3 biological replicates). DMSO controls were the same as in Supplementary Fig. 8a-d. P values were obtained using Student's t-test (unpaired two-tailed, equal variance). Source data are provided as a Source Data file.
