# Supplementary material for: Mitotic microhomology-mediated break-induced replication promotes chromoanasynthesis
Source: Nat Commun. 2026 Mar 3;17:3375. doi: 10.1038/s41467-026-70086-y (PMC13065848; doi:10.1038/s41467-026-70086-y)
Supplement: Supplementary file 2 — Description of Additional Supplementary Files [file 41467_2026_70086_MOESM2_ESM.pdf]

Description of Additional Supplementary Files

File Name: Supplementary Data 1

Description: Alignment of DNA segments within complex 18 to a reference genome (T2T-CHM13v2.0) using fslr. chrom = mapped chromosome, rstart = start point at mapped chromosomes, rend = end point at mapped chromosomes, n_alignments = number of alignments, aln_size = size of alignment, qstart = start point at DNA molecule, qend = end point at DNA molecule, strand = DNA orientation, qlen = length of DNA molecule, seq = DNA sequence.

File Name: Supplementary Data 2

Description: Alignment of DNA segments within complex 12 to a reference genome (T2T-CHM13v2.0) using fslr. chrom = mapped chromosome, rstart = start point at mapped chromosomes, rend = end point at mapped chromosomes, n_alignments = number of alignments, aln_size = size of alignment, qstart = start point at DNA molecule, qend = end point at DNA molecule, strand = DNA orientation, qlen = length of DNA molecule, seq = DNA sequence.

File Name: Supplementary Data 3

Description: Alignment of DNA segments within complex 13 to a reference genome (T2T-CHM13v2.0) using fslr. chrom = mapped chromosome, rstart = start point at mapped chromosomes, rend = end point at mapped chromosomes, n_alignments = number of alignments, aln_size = size of alignment, qstart = start point at DNA molecule, qend = end point at DNA molecule, strand = DNA orientation, qlen = length of DNA molecule, seq = DNA sequence.

File Name: Supplementary Data 4

Description: Alignment of DNA segments within complex 111 to a reference genome (T2T-CHM13v2.0) using fslr and manual gap filling using BLAST (NCBI). chrom = mapped chromosome, rstart = start point at mapped chromosomes, rend = end point at mapped chromosomes, n_alignments = number of alignments, aln_size = size of alignment, qstart = start point at DNA molecule, qend = end point at DNA molecule, strand = DNA orientation, qlen = length of DNA molecule, seq = DNA sequence.

File Name: Supplementary Data 5

Description: Insertion hotspots within complex 111. chrom = mapped chromosome, rstart = start point at mapped chromosomes, rend = end point at mapped chromosomes, strand = DNA orientation.

File Name: Supplementary Data 6

Description: Alignment of DNA segments within complex 56 to a reference genome (T2T-CHM13v2.0) using fslr. chrom = mapped chromosome, rstart = start point at mapped chromosomes, rend = end point at mapped chromosomes, n_alignments = number of alignments, aln_size = size of alignment, qstart = start point at DNA molecule, qend = end point at DNA molecule, strand = DNA orientation, qlen = length of DNA molecule, seq = DNA sequence.

File Name: Supplementary Data 7

Description: Alignment of DNA segments within complex 34 to a reference genome (T2T-CHM13v2.0) using fslr. chrom = mapped chromosome, rstart = start point at mapped chromosomes, rend = end point at mapped chromosomes, n_alignments = number of alignments, aln_size = size of alignment, qstart = start point at DNA molecule, qend = end point at DNA molecule, strand = DNA orientation, qlen = length of DNA molecule, seq = DNA sequence.

File Name: Supplementary Data 8

Description: Alignment of DNA segments within complex 6 to a reference genome (T2T-CHM13v2.0) using fslr. chrom = mapped chromosome, rstart = start point at mapped chromosomes, rend = end point at mapped chromosomes, n_alignments = number of alignments, aln_size = size of alignment, qstart = start point at DNA molecule, qend = end point at DNA molecule, strand = DNA orientation, qlen = length of DNA molecule, seq = DNA sequence.

File Name: Supplementary Data 9

Description: Alignment of DNA segments within complex 21 to a reference genome (T2T-CHM13v2.0) using fslr. chrom = mapped chromosome, rstart = start point at mapped chromosomes, rend = end point at mapped chromosomes, n_alignments = number of alignments, aln_size = size of alignment, qstart = start point at DNA molecule, qend = end point at DNA molecule, strand = DNA orientation, qlen = length of DNA molecule, seq = DNA sequence.

File Name: Supplementary Data 10

Description: Alignment of DNA segments within complex 134 to a reference genome (T2T-CHM13v2.0) using fslr and manual gap filling using BLAST (NCBI). chrom = mapped chromosome, rstart = start point at mapped chromosomes, rend = end point at mapped chromosomes, n_alignments = number of alignments, aln_size = size of alignment, qstart = start point at DNA molecule, qend = end point at DNA molecule, strand = DNA orientation, qlen = length of DNA molecule, seq = DNA sequence.

File Name: Supplementary Data 11

Description: Alignment of DNA segments within complex 10 to a reference genome (T2T-CHM13v2.0) using fslr. chrom = mapped chromosome, rstart = start point at mapped chromosomes, rend = end point at mapped chromosomes, n_alignments = number of alignments, aln_size = size of alignment, qstart = start point at DNA molecule, qend = end point at DNA molecule, strand = DNA orientation, qlen = length of DNA molecule, seq = DNA sequence.

File Name: Supplementary Data 12

Description: Alignment of DNA segments within complex 9 to a reference genome (T2T-CHM13v2.0) using fslr. chrom = mapped chromosome, rstart = start point at mapped chromosomes, rend = end point at mapped chromosomes, n_alignments = number of alignments, aln_size = size of alignment, qstart = start point at DNA molecule, qend = end point at DNA molecule, strand = DNA orientation, qlen = length of DNA molecule, seq = DNA sequence.
