# Supplementary material for: Mitotic microhomology-mediated break-induced replication promotes chromoanasynthesis
Source: Nat Commun. 2026 Mar 3;17:3375. doi: 10.1038/s41467-026-70086-y (PMC13065848; doi:10.1038/s41467-026-70086-y)
Supplement: Supplementary file 15 — Reporting Summary [file 41467_2026_70086_MOESM15_ESM.pdf]

nature portfolio

Corresponding author(s): Greg Ngo, Duncan Baird

Last updated by author(s): Feb 11, 2026

Reporting Summary

Nature Portfolio wishes to improve the reproducibility of the work that we publish. This form provides structure for consistency and transparency in reporting. For further information on Nature Portfolio policies, see our Editorial Policies and the Editorial Policy Checklist.

Statistics

For all statistical analyses, confirm that the following items are present in the figure legend, table legend, main text, or Methods section.

n/a	Confirmed	
☐	☒	The exact sample size (*n*) for each experimental group/condition, given as a discrete number and unit of measurement
☐	☒	A statement on whether measurements were taken from distinct samples or whether the same sample was measured repeatedly
☐	☒	The statistical test(s) used AND whether they are one- or two-sided<br>*Only common tests should be described solely by name; describe more complex techniques in the Methods section.*
☒	☐	A description of all covariates tested
☒	☐	A description of any assumptions or corrections, such as tests of normality and adjustment for multiple comparisons
☐	☒	A full description of the statistical parameters including central tendency (e.g. means) or other basic estimates (e.g. regression coefficient) AND variation (e.g. standard deviation) or associated estimates of uncertainty (e.g. confidence intervals)
☐	☒	For null hypothesis testing, the test statistic (e.g. *F*, *t*, *r*) with confidence intervals, effect sizes, degrees of freedom and *P* value noted<br>*Give P values as exact values whenever suitable.*
☒	☐	For Bayesian analysis, information on the choice of priors and Markov chain Monte Carlo settings
☒	☐	For hierarchical and complex designs, identification of the appropriate level for tests and full reporting of outcomes
☒	☐	Estimates of effect sizes (e.g. Cohen's *d*, Pearson's *r*), indicating how they were calculated

Our web collection on statistics for biologists contains articles on many of the points above.

Software and code

Policy information about availability of computer code

Data collection	Typhoon FLA 9500 v1.1 (GE Healthcare)<br>minKNOW v 23.07.15 (Oxford Nanopore Technologies)<br>NC-3000™ NucleoView v2.1 (Chemometec)<br>QuantStudio v1.3 Software (Thermo Fisher Scientific)<br>Zeiss Zen v2.6, blue edition (Zeiss)
Data analysis	Microsoft Excel v 16.92<br>GraphPad Prism v 10.2.3<br>FSLR (https://github.com/kcleal/fslr) v 0.3.9<br>GW (https://github.com/kcleal/gw) v 1.1.1<br>Floreada (https://floreada.io/) v3/30/25<br>QuantStudio v1.3 Software (Thermo Fisher Scientific)<br>Image J (https://imagej.net/ij/index.html) v1.54

For manuscripts utilizing custom algorithms or software that are central to the research but not yet described in published literature, software must be made available to editors and reviewers. We strongly encourage code deposition in a community repository (e.g. GitHub). See the Nature Portfolio guidelines for submitting code & software for further information.

Data

Policy information about availability of data

All manuscripts must include a data availability statement. This statement should provide the following information, where applicable:

- Accession codes, unique identifiers, or web links for publicly available datasets
- A description of any restrictions on data availability
- For clinical datasets or third party data, please ensure that the statement adheres to our policy

All analysed data are included in this paper. The raw nanopore sequencing data generated in this study have been deposited in the SRA database under accession code PRJNA1194528, without access restrictions. (https://www.ncbi.nlm.nih.gov/bioproject/PRJNA1194528). All materials are available upon request (DB). Source Data are provided with this paper.

Research involving human participants, their data, or biological material

Policy information about studies with human participants or human data. See also policy information about sex, gender (identity/presentation), and sexual orientation and race, ethnicity and racism.

Reporting on sex and gender	N/A
Reporting on race, ethnicity, or other socially relevant groupings	N/A
Population characteristics	N/A
Recruitment	N/A
Ethics oversight	N/A

Note that full information on the approval of the study protocol must also be provided in the manuscript.

Field-specific reporting

Please select the one below that is the best fit for your research. If you are not sure, read the appropriate sections before making your selection.

☒ Life sciences ☐ Behavioural & social sciences ☐ Ecological, evolutionary & environmental sciences

For a reference copy of the document with all sections, see nature.com/documents/nr-reporting-summary-flat.pdf

Life sciences study design

All studies must disclose on these points even when the disclosure is negative.

Sample size	Sample-size calculation were not performed. Experiments were repeated for 3-4 times, based on similar studies in the field.
Data exclusions	qPCR value for one of the triplicate reaction from a single sample were treated as outlier and excluded if it differs by >1 Ct from other duplicates or if it differs by >0.5 Ct from other duplicates with superimposable amplification curves. This serves to reduce experimental error introduced by pipetting error.<br>Nanopore sequencing reads harbouring probable sequencing artefacts, including long stretches of very low complexity bases and concatemers were discarded during FSLR analysis.
Replication	The experiments were repeated for the indicated number of times to confirm reproducibility. All attempts at replication were successful.
Randomization	Cells were randomly allocated for the modulation of gene activity or the induction of DNA damage.
Blinding	Blinding was not performed during data collection or analysis. This is because all data were collected by machines and analysis were performed by using programs and protocols which treat all samples equally, so it is unlikely that bias can be introduced.

Reporting for specific materials, systems and methods

We require information from authors about some types of materials, experimental systems and methods used in many studies. Here, indicate whether each material, system or method listed is relevant to your study. If you are not sure if a list item applies to your research, read the appropriate section before selecting a response.

Materials & experimental systems

n/a	Involved in the study	
☐	☒	Antibodies
☐	☒	Eukaryotic cell lines
☒	☐	Palaeontology and archaeology
☒	☐	Animals and other organisms
☒	☐	Clinical data
☒	☐	Dual use research of concern
☒	☐	Plants

Methods

n/a	Involved in the study	
☒	☐	ChIP-seq
☒	☐	Flow cytometry
☒	☐	MRI-based neuroimaging

Antibodies

Antibodies used

anti-GAPDH rabbit monoclonal antibody (2118, Cell Signaling Technology), RRID:AB_561053

anti-POLD3 rabbit monoclonal antibody (A301-244A, Cambridge Bioscience), RRID:AB_890596

anti-TRF1 mouse monoclonal antibody (ab10579, Abcam), RRID:AB_2201461

anti-Phospho-Histone H2A.X (Ser139) antibody (2577, Cell Signaling Technology), RRID:AB_2118010

Validation

anti-GAPDH rabbit monoclonal antibody, clone 14C10 (2118, Cell Signaling Technology), RRID:AB_561053, validated by supplier and publications

https://www.cellsignal.com/products/primary-antibodies/gapdh-14c10-rabbit-mab/2118?srsltid=AfmBOorETsjXRPO5XoDfR-iphgIi3oK3oz_TuvBsY1_d9A8yKLtqj2dT

anti-POLD3 rabbit polyclonal antibody (A301-244A, Cambridge Bioscience) , RRID:AB_890596, validated by supplier and publications

https://www.fortislife.com/products/primary-antibodies/rabbit-anti-pold3-p66-antibody/BETHYL-A301-244

anti-TRF1 mouse monoclonal antibody, clone TRF-78 (ab10579, Abcam), RRID:AB_2201461, validated by supplier and publications

https://www.abcam.com/en-us/products/primary-antibodies/trf2-trf1-antibody-trf-78-ab10579

anti-Phospho-Histone H2A.X (Ser139) polyclonal antibody (2577, Cell Signaling Technology), RRID:AB_2118010, validated by supplier and publications

https://www.cellsignal.com/products/primary-antibodies/phospho-histone-h2a-x-ser139-antibody/2577?srsltid=AfmBOor3C2Gy7iu-7KUcpSLTIlmnAANw2l41Rw6cKZBWCyxOohYumC2C

Eukaryotic cell lines

Policy information about cell lines and Sex and Gender in Research

Cell line source(s)

RPE1-hTERT from American Type Culture Collection (ATCC), CRL-4000, female

HCT116 from American Type Culture Collection (ATCC), CCL-247, male

U2OS from American Type Culture Collection (ATCC), HTB-96, female

MRC5 from European Collection of Authenticated Cell Cultures, ECACC number = 84101801, authenticated as ATCC cell line CCL171, male

HCA2 from James Smith, Houston, USA, PMID: 8950976, male

IMR90 from Coriell Institute Cell Repository, authenticated as ATCC cell line CCL186, female

WI38 from Coriell Institute Cell Repository, authenticated as ATCC cell line CCL75, female

All knockout cell lines were generated in house.

Authentication	RPE1-hTERT, HCT116, U2OS, MRC5, IMR90 and WI38 were authenticated by ATCC cell line authentication service (STR analysis).
Mycoplasma contamination	PCR test were performed when these cell lines were received and showed that all cell lines were mycoplasma negative. Test were repeated regularly which showed that all cell lines remained mycoplasma negative during the course of this study.
Commonly misidentified lines (See ICLAC register)	none

Plants

Seed stocks	N/A
Novel plant genotypes	N/A
Authentication	N/A
